# Impact Resistance Behaviors of Carbon Fiber Fabric Reinforced Composite Laminates with Bio-Inspired Helicoidal Layups

**DOI:** 10.3390/biomimetics10080525

**Published:** 2025-08-11

**Authors:** Lizhen Du, Jiaqi Tang, Zisheng Wang, Jiacheng Zhou, Xiaoshuang Xiong, Xiang Li, Mingzhang Chen

**Affiliations:** 1 Hubei Key Laboratory of Digital Textile Equipment, Wuhan Textile University, Wuhan 430200, China; dlzay@wtu.edu.cn; 2 School of Mechanical Engineering and Automation, Wuhan Textile University, Wuhan 430200, Chinazhoujiacheng0609@126.com (J.Z.); 3 Key Laboratory of Safety of Hydrogen Energy Storage and Transportation Equipment, State Administration for Market Regulation, Beijing 100029, China; 4 Hubei Key Laboratory of Advanced Technology for Automotive Components, Wuhan University of Technology, Wuhan 430070, China; chenmingzhang@whut.edu.cn

**Keywords:** carbon fiber fabric, composite laminates, bio-inspired helicoidal layups, low velocity impact, numerical simulation

## Abstract

Carbon fiber fabric reinforced composite laminates are widely used in the automotive and aerospace components, which are prone to suffering low velocity impacts. In this paper, helicoidal layups of fabrics inspired by the Bouligand type structure of the dactyl clubs of mantis shrimp are proposed to improve the impact resistance of carbon fiber fabric reinforced composite laminates. Low velocity impact tests and finite element simulation are carried out to investigate the effect of the rotation angle of helicoidal layups on the impact damage behaviors of composite laminates, including impact force response, energy absorption characteristics and damage mechanism. Results show that the simulation results of impact force–time response, absorbed energy–time response, and damage characteristics show good agreements with the experimental results. With the increase in impact energy, the maximum value of impact force, the absorbed energy and the energy absorption ratio for all specimens are all increased. Under all impact energies, the impact damage of specimens with helicoidal layups are lower than that of specimen QI1 (rotation angle of 0°), indicating that the helical layup of woven carbon fabric can sufficiently enhance the impact resistance of the composite material. Furthermore, the impact resistance of specimen HL2 (rotation angle of 12.8°) is the best, because it demonstrates the lowest impact damage and highest impact force under all energies. This work provides a bionic design guideline for the high impact performance of carbon fiber fabric reinforced composite laminate.

## 1. Introduction

Carbon fiber fabric reinforced composite laminates are widely used in the aerospace and automotive industries due to their inherently excellent mechanical properties [1,2]. Much research has been carried out in investigating and optimizing the impact properties of carbon fiber fabric reinforced composite through numerical simulations and experimental tests [3,4,5,6,7], which are mostly focused on the design of the weaving structure of carbon fiber fabric. Over the past few decades, natural systems have proved to be an incredible resource for the design of new material structures for impact resistance applications [8,9,10,11]. Through the observation of microstructure, helicoidally laminated structures, or so-called Bouligand type structures, have been widely observed in many different organic textures and provide excellent impact resistance, which offers inspiration for the stacking sequence design of fiber composite laminates with better impact properties [12,13,14,15,16].

Many scholars have carried out research on Bouligand type structures within different microscopic structures of organisms in order to develop bio-inspired composites, and have tried to improve the mechanical properties of fiber-reinforced composites through optimizing the stacking sequence of the fiber layer based on these bio-inspired structures [12,13,14,15,16,17]. Cheng et al. [17] firstly incorporated the distinctive helicoidal microscopic structure observed in the exoskeletons of crustaceans into the design and manufacturing of bio-inspired laminated composites. They found that the flexural properties and shear strength of bio-inspired laminated composites were superior to those of conventional laminated composites according to the experimental test results, especially the bio-inspired structure with a smaller fiber rotation angle of 7.8°. Ginzburg et al. [12] applied the microstructure observed in the dactyl clubs of mantis shrimp to the development of bio-inspired helicoidal composite laminates. Based on the results of low-velocity impact experiments and numerical simulation, they concluded that the helicoidal layups were more effective at absorbing energy, while minimizing thickness failure, than conventional cross-ply and quasi-isotropic laminates. In order to further investigate the effect of fiber rotation angle on the impact performance of bio-inspired helicoidal laminate, Grunenfelder et al. [13] investigated the impact resistance and the residual strength of bio-inspired helicoidal laminate with three different fiber rotation angles and reported that the impact resistance and residual strength of samples with a medium fiber rotation angle (16.3°) or a large fiber rotation angle (25.7°) are better than those of samples with a small fiber rotation angle (7.8°). Different from the stacking sequence of fiber layer with a linear rotation angle, Jiang et al. [14] further designed helicoidally laminated composites using a non-linear rotation angle to enhance the impact resistance. The impact failure behavior of composite laminates for four types of helicoidal configurations, i.e., quasi-isotropic layup, helicoidal-recursive layup, helicoidal-exponential layup and helicoidal-semicircular layup, were investigated using finite element modeling. This revealed that helicoidal-recursive and helicoidal-exponential layups with large rotation angles can improve the impact resistance, compared with the quasi-isotropic layup. Inspired by the coupling structure of the periodic region and impact region of the dactyl club, Han et al. [15] proposed a novel dactyl-inspired laminate with a helicoidal-fiber sinusoidal-structure, which was compared with unidirectional-fiber flat laminate, helicoidal-fiber flat laminate and unidirectional-fiber sinusoidal-structure laminate using low-velocity impact tests. Their study indicated that the dactyl-inspired laminate with a helical-fiber sinusoidalstructure has the best impact resistance for the helical arrangement of fibers, preventing the propagation of cracks, the structure enhancing the buffering of impact forces. As aforementioned, previous studies have widely investigated the effects of helicoidal layups on the mechanical properties and impact behaviors of bio-inspired unidirectional fiber-reinforced composite laminates, indicating that the fiber rotation angle has a great effect on the mechanical properties and impact behaviors of bio-inspired composites. So far, the application of bio-inspired helicoidal layups within woven fabric reinforced composite laminates has been rarely reported. Therefore, in view of the advantage of helicoidal layups inspired by bionics, it is necessary to investigate the design and optimization of helicoidal layups to further improve the impact resistance of carbon fiber fabric composite laminates.

Compared with unidirectional fiber-reinforced composites, the impact damage of plain weave fabric reinforced composites is significantly different and dramatically improved by its specific fabric architectures. In order to investigate the impact damage mechanism and properties of plain woven fabric reinforced composites, low velocity impact tests and numerical simulations have been widely carried out [18]. Zhang et al. [19] investigated the low velocity impact response of fabric composite laminates with different fabric structures through experimental tests and found that serious delamination appeared in unidirectional fiber-reinforced composite laminates, while less delamination damage appeared in 2D plain-woven fabric composite laminates. Hart et al. [20] further compared the low velocity impact response and damage formation for 2D and 3D woven composite plates under different impact energies using low velocity impact tests, and proposed that the delamination length and opening of 2D woven composites presented as larger than 3D woven composites at the same impact energy. Due to the multiscale features of plain woven composite, Hou et al. [21] developed a multiscale finite element modeling method for accurate simulation of the low-velocity impact behavior of plain-woven composite, through transferring the warp and fill yarns, as well as the resin, into 0° and 90° sub-cells, and then assembling these sub-cells into an equivalent cross-ply laminate within the internal fabric structure. They concluded that the numerical results were in good agreement with the experimental tests, and the delamination and matrix-based damages were the main failure modes. Considering the plasticity and damage of plain woven composites, Gupta et al. [22] introduced an improved orthotropic elasto-plastic damage model into the simulation of low velocity impact test, which proved to be useful in predicting impact load–displacement, impact energy–time, impact deformation–time curves and impact damage zones accurately. As aforementioned, low velocity impact tests and numerical simulations have been widely used to effectively analyze the impact behaviors of plain woven fabric reinforced composites. However, most research is focused on the low velocity impact response of conventional plain woven composite with the same layups for each layer, and few studies have been carried out on plain woven composite with helicoidal layups inspired by bio-structures.

In this work, inspired by bionics, helicoidal layups are designed to improve the impact resistance capacity of plain woven carbon fiber-reinforced composite laminates. Low velocity impact tests and finite element simulations are carried out to investigate the effect of the rotation angle of helicoidal layups on the impact damage behaviors of composite laminates, including impact force response, energy absorption characteristics and damage mechanism. Through detailed analysis, the optimal rotation angle of the bionic helicoidal layup is obtained. This work provides a bionic design guideline for high impact performance of plain woven composite laminate.

## 2. Experiment

### 2.1. Bio-Inspired Helicoidal Composite Laminate Design

**Figure 1:**
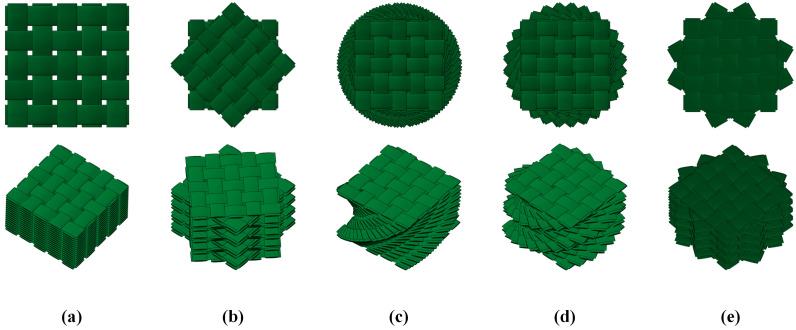
Schematic diagram of 5 types of helicoidal layups: (**a**) QI1, (**b**) QI2, (**c**) HL1, (**d**) HL2, and (**e**) HL3.

A reasonable choice of layup configuration is fairly critical to the design of bio-inspired composite laminates. Numerous studies have been carried out on the rotation angle of bio-inspired composite laminates, in which the rotation angle ranged from 6° to 25.7° and was kept equal between each layer [12,13,14,15,16,17,23,24,25]. According to the above referenced rotation angle, we propose a helicoidal composite lamination scheme and adopt a linear rotation angle per helicoidal layer. Considering the mathematical relationship between the number of layers and repetition times, three types of bio-inspired composite laminates consisted of 16 layers of plain woven carbon fabric prepreg (weaving angle of 0–90°) arranged with a linear rotation angle per ply are presented in Table 1 and Figure 1. Furthermore, two conventional quasi-isotropic composite laminates were used to contrast with the three bio-inspired composite laminates.

### 2.2. Materials and Processing

In order to investigate the impact performance of bio-inspired composite laminates, five types of composite laminates were made by plain woven carbon fabric prepregs (weaving angle of 0/90°) provided by Yixing Zhongfu Carbon Fiber Products Co., Ltd (Yixing, China). The plain woven carbon fabric prepreg with an average areal density of 200 g/m^2^ contains carbon fibers T300/3K (density: 1.78 g/cm^3^, diameter: 7.0 μm) and epoxy resin 7901 (density: 1.20 g/cm^3^), their mechanical properties listed in Table 2.

**Figure 2:**
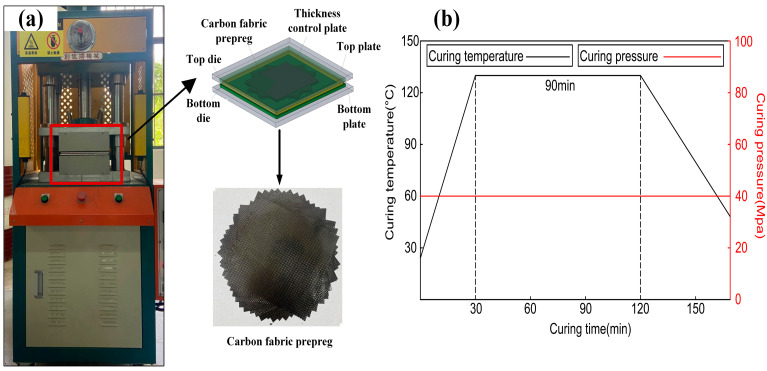
Schematic diagram of composite laminated plate processing: (**a**) materials and equipments and (**b**) parameters of hot pressing process.

Firstly, 16 pieces of plain woven carbon fabric prepreg of 175 mm × 175 mm × 0.20 mm were laid in an arrangement with the pre-set rotation angle per ply, as shown in Figure 2a. Secondly, the laid plain woven carbon fabric prepreg were put into a hot press for hot forming, as presented in Figure 2a. As shown in Figure 2b, the hot pressure and temperature were set to 40 MPa and 130 °C, respectively. The total duration of the hot pressing process was approximately 2 h. Next, the heating device was turned off and the laminate cooled to room temperature under a pressure of 40 MPa. The composite laminates were finally cut into a standard size of 120 mm × 120 mm × 3 mm for the low-velocity impact test.

### 2.3. Low-Velocity Impact Test

**Figure 3:**
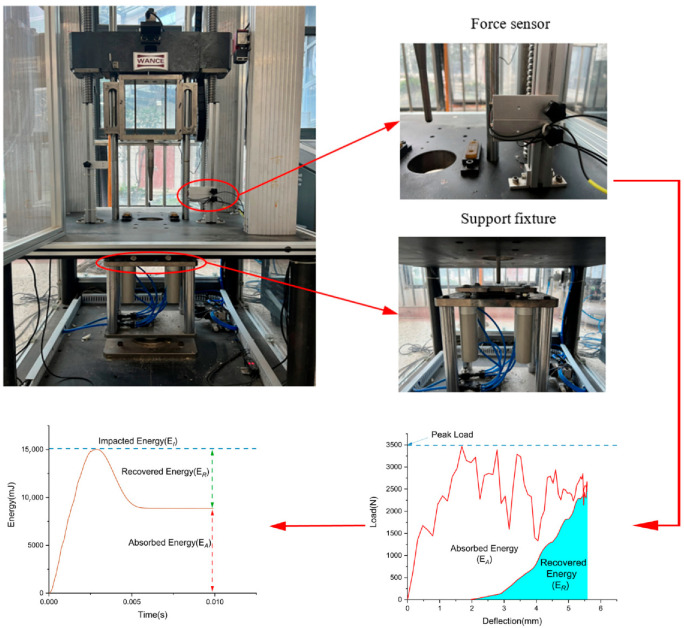
Schematic of low-velocity impact experiment including the collection of force and energy data.

In the present study, the low velocity impact tests were carried out using a drop weight machine based on the ASTM D7136, which is shown in Figure 3 [26]. As presented in Figure 3, a hemispherical impactor weighing 4 kg and with a diameter of 12 mm was used in the tests, equipped with force sensors to measure the contact force between the impactor and specimens. The impacts occurred at the center of the specimens, which were clamped by two fixtures (100 mm × 100 mm × 5 mm) with a central hole with a diameter of 8 cm. The force data obtained from output of force sensor are recorded digitally and then used to calculate the energy data, as shown in Figure 3. The energy absorbed by the specimen is mostly dissipated in deformation and damage, equal to the change in kinetic energy of the impactor. As listed in Table 3, the specimens were tested at three impact energies (5 J, 10 J and 15 J) referring to the literature [22], while each test at the same impact energy was repeated three times. After the impact test, all the specimens were checked for impact damage on both the impacted and non-impacted surfaces using a digital camera. To evaluate the extent of impact damage along the thickness of specimen, the ultrasonic A-scans were executed using a 20 MHz probe.

## 3. Numerical Model

### 3.1. Finite Element Modeling

**Figure 4:**
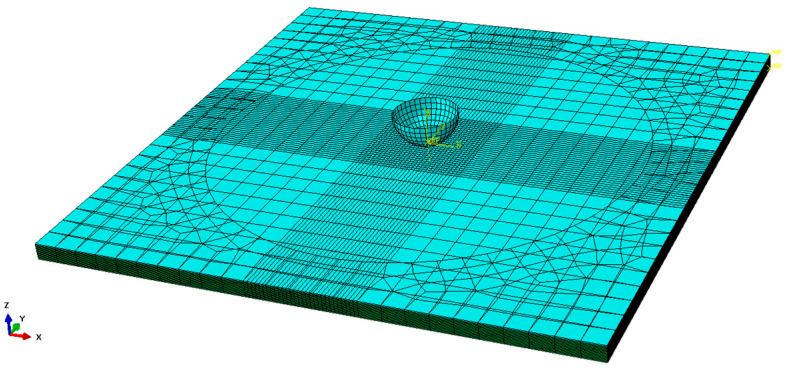
Finite element modeling of composite laminates for low-velocity impact testing.

In order to predict the impact properties and investigate the failure modes of bio-inspired helicoidal composite laminates, a 3D finite element (FE) model of the 100 mm × 100 mm × 3 mm laminate was developed by using the Abaqus 6.21/Explicit software to simulate low-velocity impact tests, as presented in Figure 4. In the modelling of impact test, the steel impactor was set as a hemispherical rigid body with mass of 4 kg and a diameter of 12 mm, while it was meshed with the 4-node rigid shell elements (R3D4). The friction interaction (μ = 0.15) between the impactor and the composite laminate was defined by general contact algorithm. Three initial vertical velocities were applied to the mass center of the impactor to achieve the experimental impact energies, while all the rotational degrees of freedom and the translational degrees of freedom in the direction of U_1_ and U_2_ of the mass center of the rigid impactor were constrained. The helicoidal composite laminate with 16 layers was modeled as the deformable body meshed with the 3D 8-node brick elements (C3D8R), and a single element across the thickness was used in each ply, with a thickness of 0.187 mm, as shown Figure 4. The element size of the impact region (20 mm × 20 mm in the center of each ply) was meshed with 0.5 mm × 0.5 mm along the length and width directions, and the far region was coarsely meshed. Cohesive elements (COH3D8) were used to mesh each interlayer interface (15 layers), with a thickness of 0.01 mm between the adjacent plies. In addition, the translational and rotational degrees of freedom of the elements in the upper and lower surfaces of the composite laminate constrained by two fixtures were fixed. Furthermore, the laying angles of different layers was achieved by setting the material orientation in the coordinate system of each layer.

### 3.2. Damage Model for the Composite Laminate

**Figure 5:**
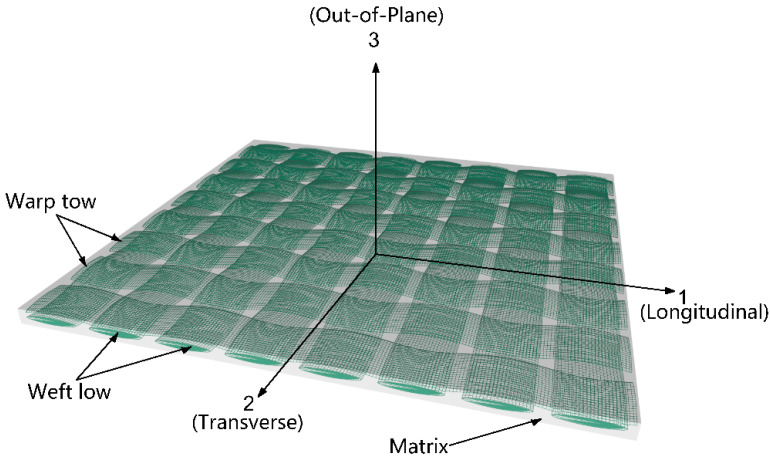
Schematic diagram of single lamina of composite laminate.

Each lamina of the composite laminate can be regarded as an orthotropic material with three principal material directions, which are the warp, weft and out-of-plane directions. The material relationship between the stress and strain is defined in the form of the compliance matrix, *S*_0_:

(1) $$\varepsilon =S_{0}\sigma$$

where *ε*, *S*_0_ and *σ* are the engineering strain matrix, the compliance matrix and the stress matrix, respectively. The compliance matrix *S*_0_ without damage can be written as Equation (2) [22]:

(2) $$S_{0}=\left[\begin{array}{cccccc} \frac{1}{E_{11}} & -\frac{\upsilon_{21}}{E_{22}} & -\frac{\upsilon_{31}}{E_{33}} & 0 & 0 & 0\\ -\frac{\upsilon_{12}}{E_{11}} & \frac{1}{E_{22}} & -\frac{\upsilon_{32}}{E_{33}} & 0 & 0 & 0\\ -\frac{\upsilon_{13}}{E_{11}} & -\frac{\upsilon_{23}}{E_{22}} & \frac{1}{E_{33}} & 0 & 0 & 0\\ 0 & 0 & 0 & \frac{1}{G_{12}} & 0 & 0\\ 0 & 0 & 0 & 0 & \frac{1}{G_{13}} & 0\\ 0 & 0 & 0 & 0 & 0 & \frac{1}{G_{23}} \end{array}\right]$$

where *E_ii_*, *G_ij_* and *ν_ij_* (*i*, *j* = 1, 2, 3) are the elastic modulus, the shear modulus and the Poisson ratio, respectively. 1, 2, 3 represent the warp, weft and out-of-plane direction, respectively, as presented in Figure 5. Here, $\frac{\upsilon_{ij}}{E_{ii}}=\frac{\upsilon_{ji}}{E_{jj}}$, $E_{11}=E_{22}$ and $G_{13}=G_{23}$.

The above material model of the lamina of the composite laminate is an undamaged model. When damage occurs, the continuum damage model (CDM) is then used to calculate the degraded stiffness.

#### 3.2.1. Intralaminar Damage Model

In the continuum damage model of the intralaminar material, six intralaminar damage variables further depend upon the different damage modes, which can be obtained as the following equations [22,27]:

Mode 1: *R*_1T_ for tension failure in the warp direction:

(3) $$R_{1T}={\left(\frac{\sigma_{e11}}{S_{1T}}\right)}^{2}+{\left(\frac{\sigma_{e13}}{S_{13}}\right)}^{2}$$

Mode 2: *R*_1C_ for compression failure in the warp direction:

(4) $$R_{1C}={\left(\frac{\sigma_{e11}}{S_{1C}}\right)}^{2}$$

Mode 3: *R*_2T_ for tension failure in the weft direction:

(5) $$R_{2T}={\left(\frac{\sigma_{e22}}{S_{2T}}\right)}^{2}+{\left(\frac{\sigma_{e23}}{S_{23}}\right)}^{2}$$

Mode 4: *R*_2C_ for compression failure in the weft direction:

(6) $$R_{2C}={\left(\frac{\sigma_{e22}}{S_{2C}}\right)}^{2}$$

Mode 5: *R*_3C_ for compression failure in the out-of-plane direction:

(7) $$R_{3C}={\left(\frac{\sigma_{e33}}{S_{3C}}\right)}^{2}$$

Mode 6: *R*_12_ for in-plane shear failure:

(8) $$R_{12}={\left(\frac{\sigma_{e12}}{S_{12}}\right)}^{2}$$

where the failure factor *R*_i_ (i = 1T, 1C, 2T, 2C, 3C, 12) represents the degree of damage; *S*_1T_ and *S*_1C_ are warp direction tensile and compressive strengths; *S*_2T_ and *S*_2C_ are weft direction tensile and compressive strengths; *S*_3C_ is out-of-plane compressive strength; *S*_13_ and *S*_23_ are out-of-plane shear strengths; *S*_12_ is in-plane shear strength. When the failure factor $R_{i}>1$, damage starts to occur and then the stiffness of the material decreases. Then, the damage variable $\omega_{i}$ is defined to reflect the damage degree of the material as in the following equation [28]:

(9) $$\omega_{i}=1-\exp\left(\frac{1}{m_{i}e}{\left(1-R_{i}\right)}^{m_{i}}\right)\;\;\;\;\;\;\;i\in \left\{\left.1T,\;1C,\;2T,\;2C,\;3C,\;12\right\}\right.$$

where *m*_i_ is the material softening parameter for failure mode ‘*i*’, and ‘*e*’ is the Napier’s constant.

Taking into account the coupling between the failure modes, the relationship between the damage variable $\omega_{i}$ and stiffness reduction factor *D*_ii_ can be expressed as follows [17]:

(10) $$\left\{\begin{array}{c} D_{11}=\max\left[\left(1-\left(1-\omega_{1T}\right)\left(1-\omega_{1C}\right)\right),\;\left(\omega_{3C}\right)\right]\\ D_{22}=\max\left[\left(1-\left(1-\omega_{2T}\right)\left(1-\omega_{2C}\right)\right),\;\left(\omega_{3C}\right)\right]\\ D_{33}=\left[\omega_{3C}\right]\\ D_{44}=\max\left[\left(1-\left(1-\omega_{1T}\right)\left(1-\omega_{1C}\right)\right),\;\left(1-\left(1-\omega_{1T}\right)\left(1-\omega_{1C}\right)\right),\left(\omega_{3C}\right)\right]\\ D_{55}=\max\left[\left(1-\left(1-\omega_{1T}\right)\left(1-\omega_{1C}\right)\right),\;\left(\omega_{3C}\right)\right]\\ D_{66}=\max\left[\left(1-\left(1-\omega_{2T}\right)\left(1-\omega_{2C}\right)\right),\;\left(\omega_{3C}\right)\right] \end{array}\right.$$

When damage starts to occur, the constitutive relationship of stress and strain should be updated according to the degradation of the stiffness matrix. The relationship between the damage compliance matrix *S*_d_ and the compliance matrix *S*_0_ can be expressed as follows:

(11) $$S_{d}=\left[\begin{array}{cccccc} \frac{1}{1-D_{11}} & 0 & 0 & 0 & 0 & 0\\ 0 & \frac{1}{1-D_{22}} & 0 & 0 & 0 & 0\\ 0 & 0 & \frac{1}{1-D_{33}} & 0 & 0 & 0\\ 0 & 0 & 0 & \frac{1}{1-D_{44}} & 0 & 0\\ 0 & 0 & 0 & 0 & \frac{1}{1-D_{55}} & 0\\ 0 & 0 & 0 & 0 & 0 & \frac{1}{1-D_{66}} \end{array}\right]S_{0}$$

A VUMAT sub-routine describing the aforementioned damage model has been implemented in ABAQUS/Explicit and the mechanical properties of the plain woven carbon fabric prepreg in Table 2 are assigned to the initial compliance matrix *S*_0_.

#### 3.2.2. Interlaminar Damage Using Cohesive Surfaces

A cohesive zone model (CZM) is adopted to capture the delamination between adjacent plies of the plain woven composites. The CZM approach follows a traction–separation law, in which the delamination initiation and evolution are determined by the traction stresses and separation displacements of the nodes within cohesive elements. Details of the corresponding damage criteria and evolution law are given in Equations (12)–(14) [22,26,29]:

(12) $$t=\left(\begin{array}{c} t_{n}\\ t_{s}\\ t_{t} \end{array}\right)=\left[\begin{array}{ccc} k_{n} & & \\ & k_{s} & \\ & & k_{t} \end{array}\right]\left(\begin{array}{c} \delta_{n}\\ \delta_{s}\\ \delta_{t} \end{array}\right)=K\delta$$

where *t*, *K* and *δ* are the nominal traction stress vector, stiffness vector and separation displacement vector, respectively. *n* represents the normal (out-of-plane) direction, and *s* and *t* represent two shear (in-plane) directions:

(13) $$K_{i}=\frac{C_{i}}{10t_{e}}\;\;i\in \left\{33,31,32\right\}$$

where *C_i_* represents the out-of-plane stiffness (*E*_33_) and the in-plane stiffness (*G*_31_ and *G*_32_), and *t_e_* is the element thickness of the adjacent layers.

The mixed-mode relationship proposed by the Benzeggagh and Kenane (B–K) criterion [29] was adopted to determine delamination propagation:

(14) $$\left\{\begin{array}{c} G_{n}^{C}+\left(G_{s}^{C}-G_{n}^{C}\right){\left\{\frac{G_{S}}{G_{T}}\right\}}^{\eta }=G^{C}\\ \begin{array}{l} G_{S}=G_{ss}+G_{tt} \end{array}\\ \begin{array}{l} G_{T}=G_{nn}+G_{ss}+G_{tt} \end{array} \end{array}\right.$$

where $G_{nn}$, $G_{ss}$, and $G_{tt}$ denote the dissipated energy in the normal and shear directions, respectively. $G_{n}^{C}$ and $G_{s}^{C}$ are the critical fracture energy of mode-I in the normal direction and critical fracture energy of mode-II in the shear direction, respectively, and $G_{n}^{C}$ = 0.6 N/mm, $G_{s}^{C}$ = 2.1 N/mm. *G^C^* is the critical fracture energy of the mixed-mode. Moreover, η is the B–K material coefficient, setting to 1.45.

## 4. Results and Discussion

In this section, the validity of the FE model is verified by comparing the numerical simulation results with the results of low-velocity impact test in terms of impact force, absorbed energy, and damage characteristics. The impact damage mechanism of the composite laminates is investigated based on the numerical simulation results.

### 4.1. Impact Load Response

**Figure 6:**
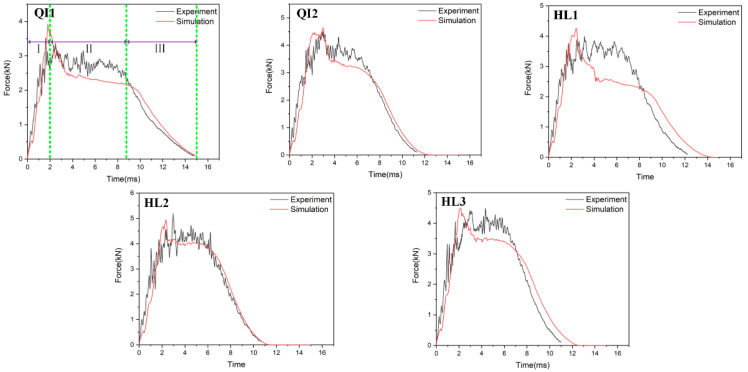
Impact force–time curves of five types of composite laminates with different helical ply angles under the impact energy of 5 J.

**Figure 7:**
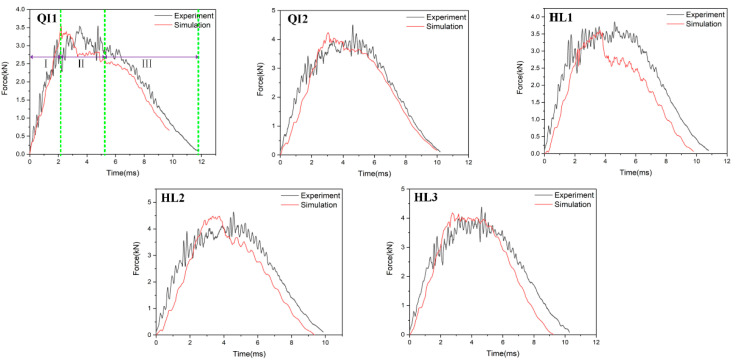
Impact force–time curves of five types of composite laminates with different helical ply angles under the impact energy of 10 J.

**Figure 8:**
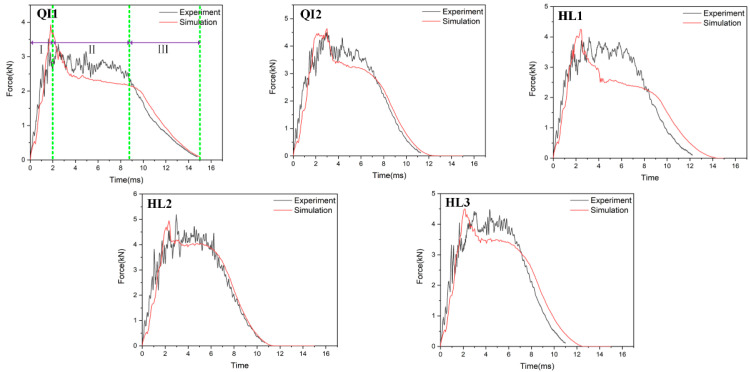
Impact force–time curves of five types of composite laminates with different helical ply angles under the impact energy of 15 J.

Figure 6, Figure 7 and Figure 8, respectively, compare the numerical results of the impact force–time curves of all the composite laminated plates under impact energies of 5 J, 10 J and 15 J with the experimental results. As shown in Figure 6, Figure 7 and Figure 8, the numerical and experimental curves under all impact energy present a similar tendency and can be divided into three stages, corresponding to the different stages of the impact procedure. In the first stage (Stage Ⅰ), the impact force increases rapidly as the contact occurs between the impactor and the specimen, indicating no or little damage of the specimen. In Stage Ⅱ, when the impact force increases to a high level, the curves exhibit an oscillatory fluctuation with a gradually decreasing trend, which is attributed to the damage propagation within each specimen. As shown in Stage Ⅲ, the kinetic energy is completely dissipated and the impact force declines dramatically with the rebound of the impactor. Similar results have been presented in the literature [16,30,31,32]. With the increase in impact energy, the impact force–time curves of all specimens in the Stage Ⅱ increasingly fluctuate, and also increase, because of the higher impact damage under higher impact energy.

In order to verify the accuracy of the FE model, the maximum values of the impact force obtained from the numerical results and experimental results are listed in Table 4. It can be seen that the average error of the maximum value of the impact force of all condition ranges from 0.2% to 23.3%, indicating that the numerical simulations are in good agreement with the experimental tests. With the increase in impact energy, both the experimental and numerical maximum values of the impact force of all specimens are increased. Under all impact energy conditions, it can be distinctly observed that the maximum value of impact force of specimen HL2 (rotation angle of 12.8°) is higher than that of the other specimens, while the maximum value of impact force of specimen QI1 (rotation angle of 0°) is much lower than that of the other specimens. Furthermore, the maximum value of impact force of specimen HL3 (rotation angle of 6°) is slightly lower than that of HL2. This indicates that the bio-inspired helicoidal layer results in a higher impact resistance performance for composite laminate compared to the non-rotation specimen. This is because continuous matrix cracking between the adjacent plies along the thickness direction is redirected along with the gradual and helicoidal change of woven fabric angle between each ply. Obviously, the HL2 exhibits the best impact resistance performance, following the HL3, and the impact resistance performance of QI1 is the worst.

**Figure 9:**
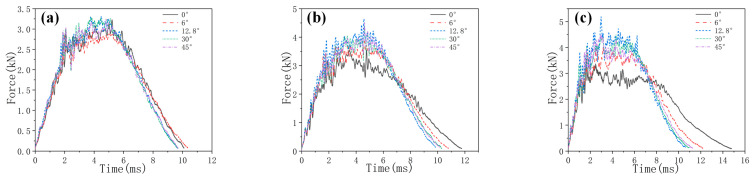
Experimental results of impact force-time curves of five types of composite laminate with different helical ply angles under different impact energies: (**a**) 5 J, (**b**) 10 J and (**c**) 15 J.

Additionally, Figure 9 compares the experimental results of impact force–time curves of the composite laminates under the three impact energies to investigate the effect of the rotation angle. It can be found that Stage Ⅱ of the specimen QI1 (rotation angle of 0°) changes greatly with the increase in impact energy and is much lower than all the other rotation angle specimens at a high impact energy of 15 J, indicating once more the higher impact damage and lower impact resistance performance of QI1 compared to other specimens.

### 4.2. Energy Absorption

**Figure 10:**
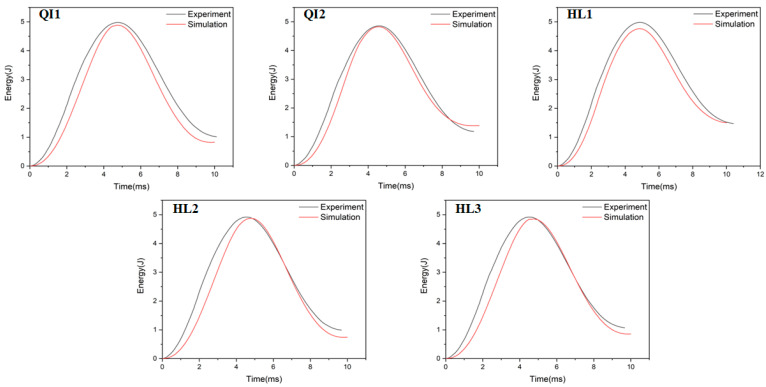
Absorbed energy–time curves of five types of CFRP laminate under the impact energy of 5 J.

**Figure 11:**
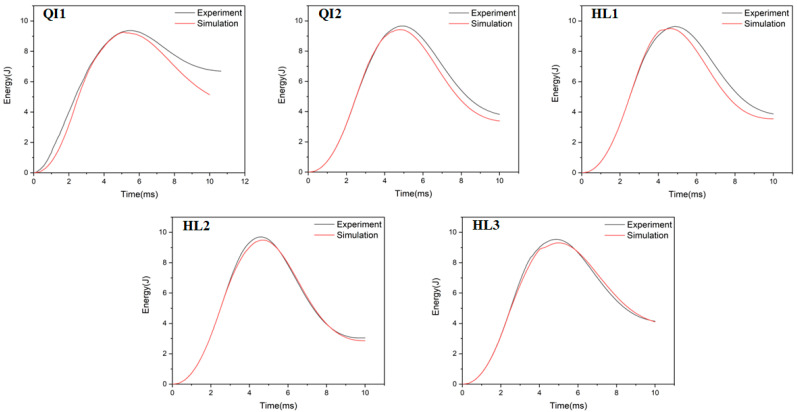
Absorbed energy–time curves of five types of CFRP laminate under the impact energy of 10 J.

**Figure 12:**
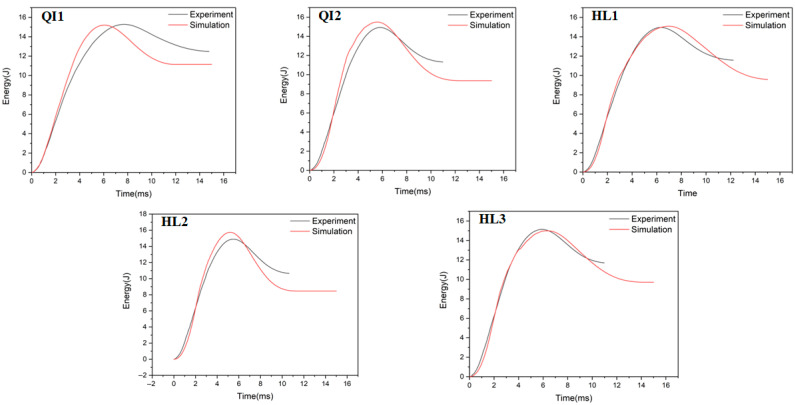
Absorbed energy–time curves of five types of CFRP laminate under the impact energy of 15 J.

The absorbed energy–time curves of the composite laminates between the numerical and experimental results obtained under the impact energies of 5 J, 10 J and 15 J are presented in Figure 10, Figure 11 and Figure 12, respectively. As presented in Figure 10, Figure 11 and Figure 12, the numerical and experimental curves for absorbed energy show good agreement. The numerical and experimental results of absorbed energy under different impact energy are listed in Table 5. The average error between the numerical and experimental results of absorbed energy ranges from 1.3% to 22.7%, again verifying the accuracy of the simulation model. The absorbed energy of the specimen generally consists of the elastic energy and the dissipated energy [26]. For all specimens, the absorbed energy is increased with the increase in impact energy, as well as the corresponding energy absorption ratio. This is because the predominant failure mode is matrix deformation under low impact energy, and this becomes fiber breakage under high impact energy [33,34]. Under low impact energy of 5 J, the energy absorption ratio is ranked as HL1 > QI2 > HL3 > QI1 > HL2, while the energy absorption ratio is ranked as QI1 > HL3 > HL1 > QI2 > HL2 under high impact energies of 10 J and 15 J.

**Figure 13:**
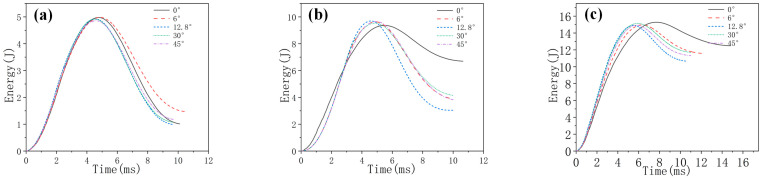
Experimental results of absorbed energy–time curves of five types of composite laminate with different helical ply angles under different impact energies: (**a**) 5 J, (**b**) 10 J and (**c**) 15 J.

Figure 13 compares the experimental results of the absorbed energy–time curves for the composite laminates under three impact energies to investigate the effect of the rotation angle. Under the low impact energy of 5 J, the energy absorption ratio of all specimens shows no significant difference, while the energy absorption ratio of HL1 is the highest and reaches 29.6%. When the impact energy increases to10 J, the energy absorption ratio of all specimens except QI1 increases slightly and reaches about 35%, while the energy absorption ratio of QI1 increases significantly to 66.9%. As is well known, the main energy absorption mechanism for the fiber-reinforced composite laminate under impact load is impact damage, including fiber fracture and matrix cracking [35]. Therefore, this indicates that a higher degree of impact damage appears in QI1 under impact energy increases to 10 J, resulting in a higher energy absorption ratio. With the impact energy increasing to 15 J, the impact damage of all specimens greatly increases and causes the energy absorption ratio to completely exceed 70%, while the energy absorption ratio of HL2 and QI1 is 71% and 83%, respectively. It can be further inferred that specimen HL2 has the best impact resistance performance under high impact energy due to its lower level of damage, while specimen QI1 has the worst impact resistance performance due to its higher damage energy absorption ratio.

### 4.3. Impact Resistance and Damage Mechanism

**Figure 14:**
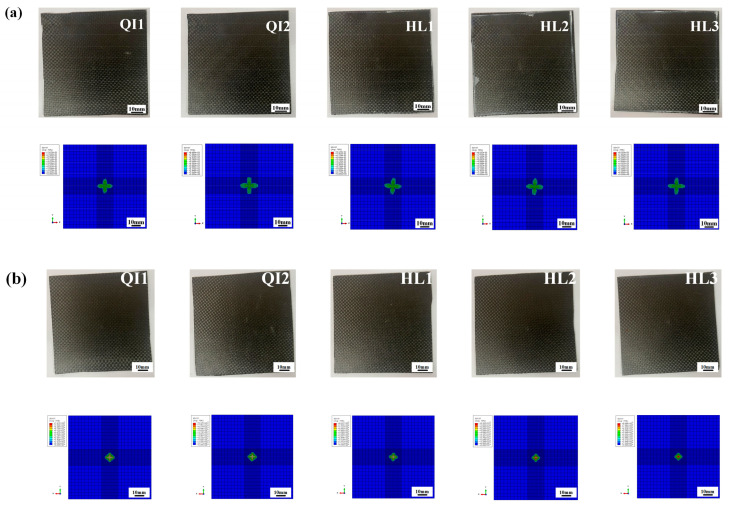
Comparison of the experimental and numerical results of the damage graphs of (**a**) impacted and (**b**) non-impacted sides for all specimens under the impact energy of 5 J.

**Figure 15:**
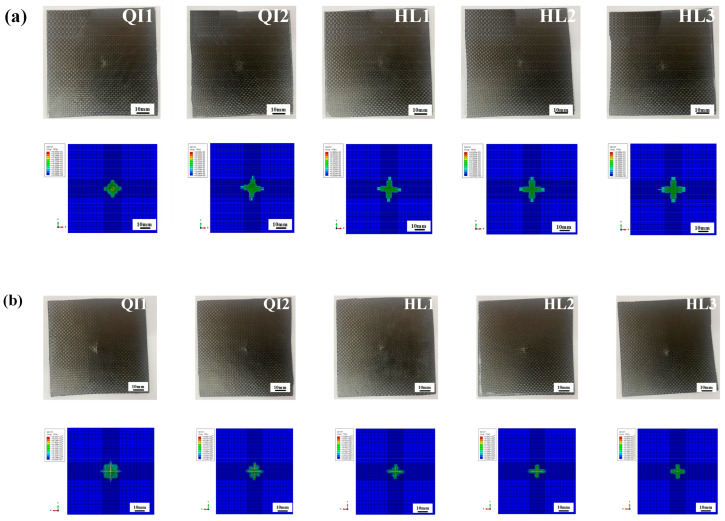
Comparison of the experimental and numerical results of the damage graphs of (**a**) impacted and (**b**) non-impacted sides for all specimens under the impact energy of 10 J.

**Figure 16:**
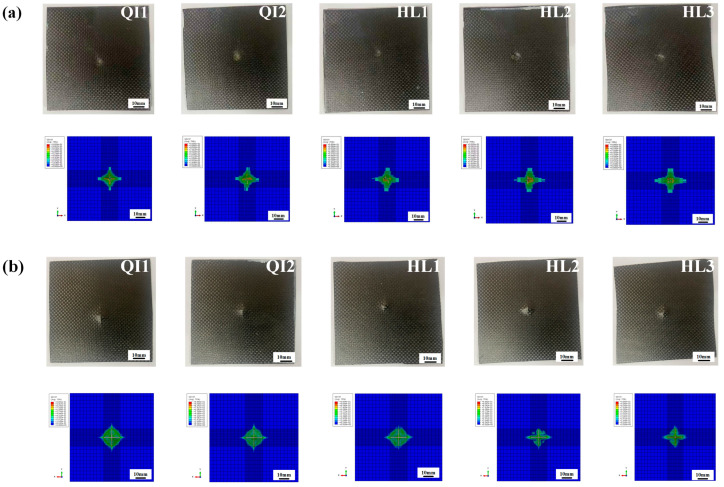
Comparison of the experimental and numerical results of the damage graphs of (**a**) impacted and (**b**) non-impacted sides for all specimens under the impact energy of 15 J.

The damage graphs for both impacted and non-impacted sides for all specimens under three impact energies obtained from the numerical simulations and experimental tests are presented in Figure 14, Figure 15 and Figure 16. Obviously, the numerical results for the macroscopic characteristics of damage on both sides for all specimens show good agreement with the experimental results. As shown in Figure 14, only slight deformation can be observed on the impacted sides, distributed around the impact point for all specimens under the impact energy of 5 J, while no obvious cracks or deformations were observed on the non-impacted side. In Figure 15, a relatively obvious dent appears on the impacted side for all specimens and no fiber breakage can be observed on the impacted side under the impact energy of 10 J. On the non-impacted side, slight cracks can be observed, indicating the fiber tensile damage and matrix breakage, corresponding to the increase in energy absorption ratio.

As shown in Figure 16, when the impact energy reaches 15 J, the dents on the impacted sides and cross-shaped cracks on the non-impacted sides for all specimens become more serious than those under the impact energy of 10 J. The distinct cross-shaped cracks on the non-impact sides for all specimens in the simulation results indicate the severe fiber tensile damage. Cross-shaped cracks appear on both sides of specimen QI1 and indicate its worst impact resistance performance, which also reveal that the high energy absorption rate of specimen QI1 is attributed to the severe impact damage. The area of cross-shaped cracks on the non-impacted sides for specimen HL2 is obviously smaller than those of other specimens, which demonstrates that the damage degree of fiber breakage and matrix crack of specimen HL2 is the least, and it has the best impact resistance performance compared with other specimens.

**Figure 17:**
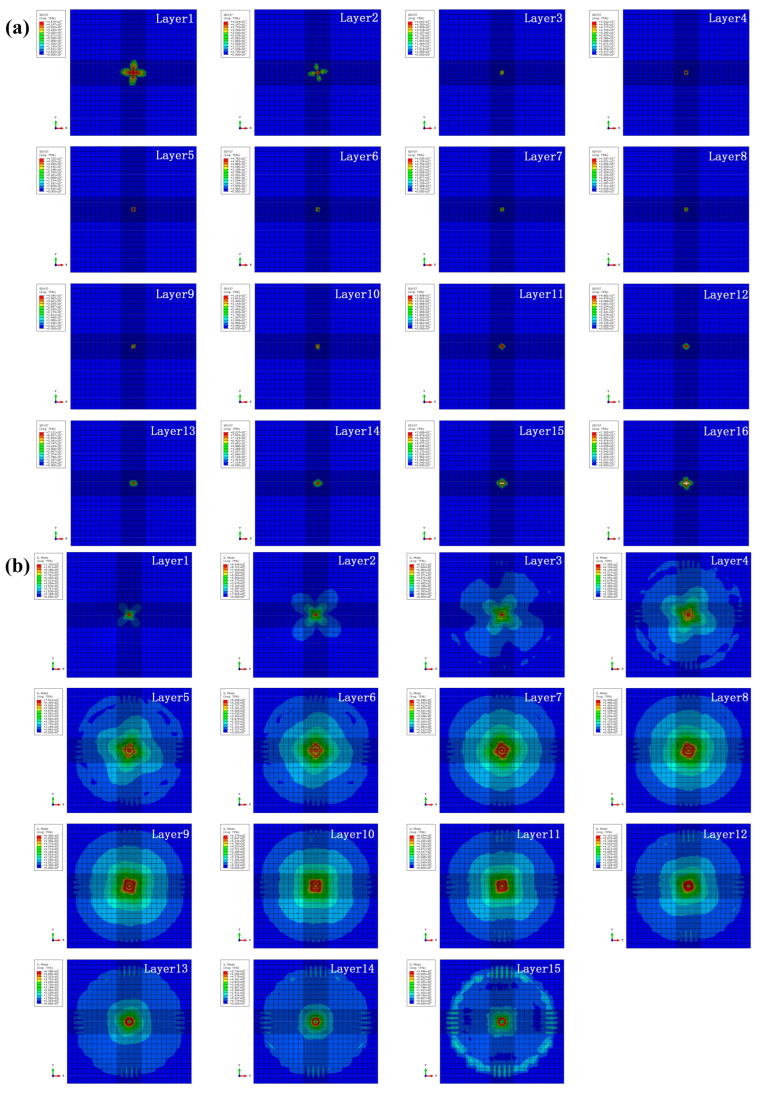
(**a**) Intralayer damage and (**b**) interlayer stress distribution for specimen HL2 under the impact energy of 5 J.

**Figure 18:**
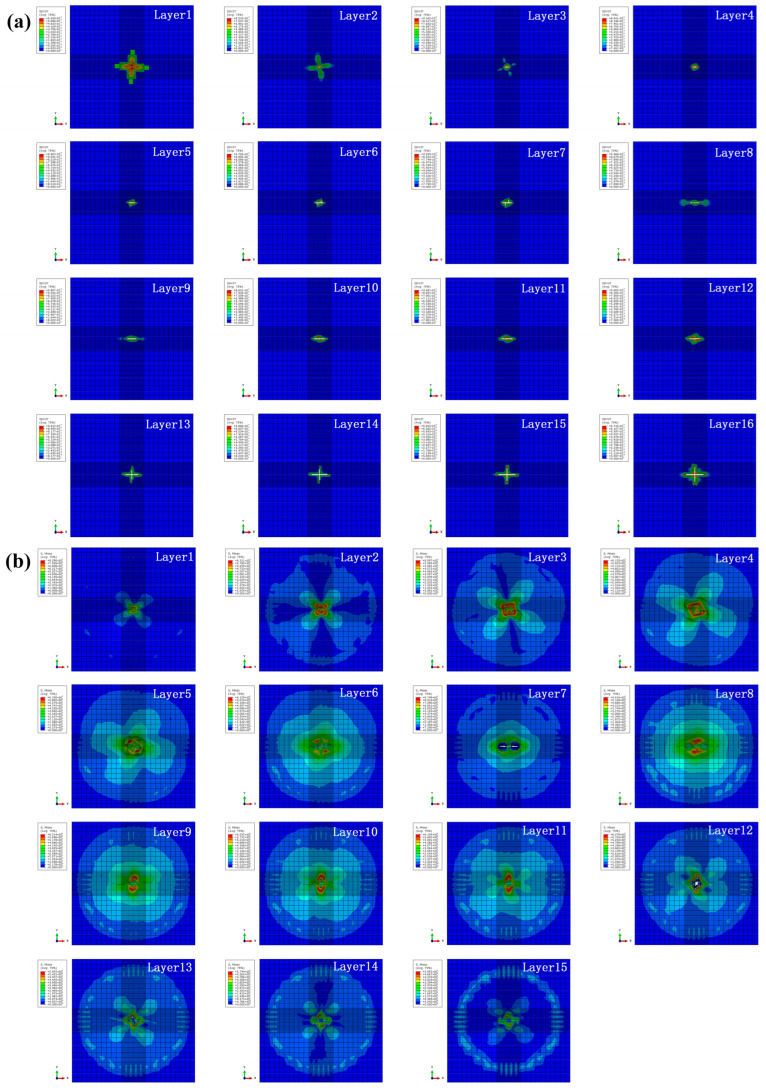
(**a**) Intralayer damage and (**b**) inter-layer stress distribution for specimen HL2 under the impact energy of 10 J.

**Figure 19:**
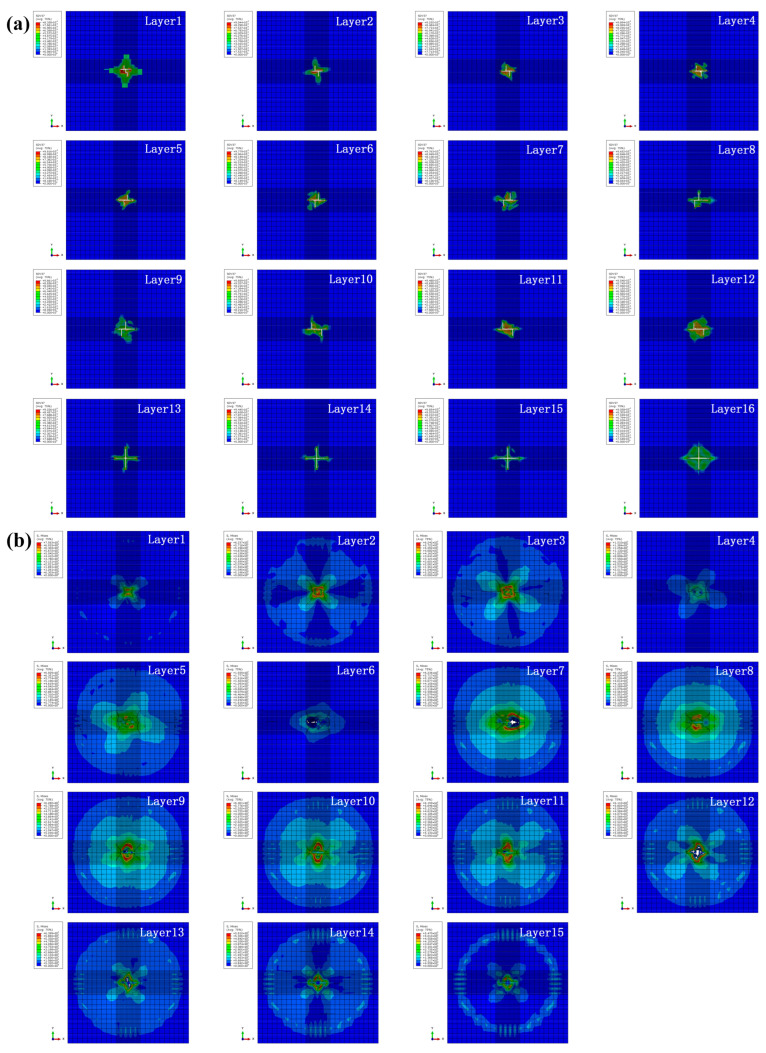
(**a**) Intralayer damage and (**b**) interlayer stress distribution for specimen HL2 under the impact energy of 15 J.

**Figure 20:**
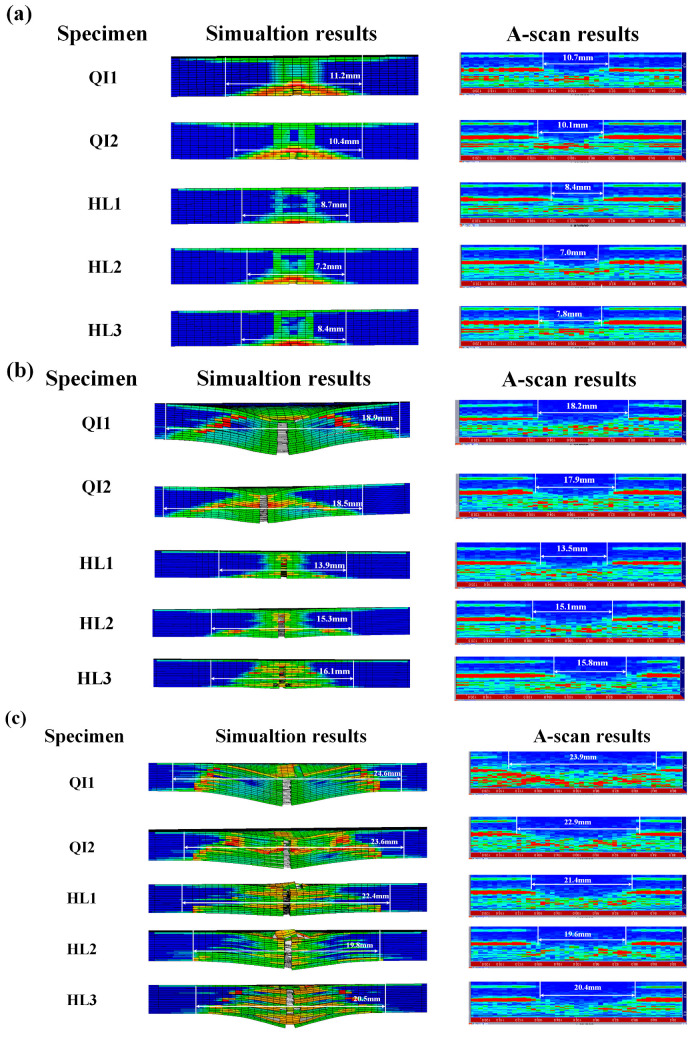
Comparison between the stress and deformation of specimens obtained from simulation results with the experimental results obtained from ultrasonic A-scan tests under different impact energies: (**a**) 5 J, (**b**) 10 J and (**c**) 15 J.

According to the above research results, it can be found that the specimen HL2 (rotation angle of 12.8°) shows the best impact resistance performance and the least macroscopic visible impact damage. Therefore, it is necessary and of importance to investigate the intralayer and interlayer damage mechanism for specimen HL2 under different impact energies, which are presented in Figure 17, Figure 18, Figure 19 and Figure 20. As shown in Figure 17a, the intralayer damage for specimen HL2 under 5 J impact energy is moderate in the middle layers and severe in the outside layers, and the overall level of damage is relatively low. Only slight cross-shaped damage appears on the first two layers. As shown in Figure 17b, the maximum value for stress decreased slightly from the impacted side to the non-impacted side under 5 J impact energy, while no obvious damage can be found on the interlayers. Furthermore, the stress deformation area of layer 8 is the largest, indicating that it suffers more bending deformation under low impact energy, showing similar results to the study of Zhao [36].

The intralayer damage and interlayer stress distribution for specimen HL2 under the impact energy of 10 J are presented in Figure 18. When the impact energy increases to 10 J, the deletion of elements can be obviously observed on some intralayers and interlayers, and explains the increase of impact damage and energy absorption ratio. Futhermore, cross-shaped cracks can be found on the last four intralayers and severe matrix damage appears on the last four interlayers, which is consistent with the impact damage morphology of the test and the increase in the energy absorption ratio under the impact energy of 10 J.

As shown in Figure 19, with the impact energy increasing to 15 J, element deletion can be observed on all intralayers and the fiber tensile damage on the non-impacted sides is more severe, indicating that the specimen is basically completely penetrated and reaches its ultimate impact ability. It can be further found that damage expansion of the intralayers along the impact direction is not continuous because of the bio-inspired structure, resulting in the improvement in impact resistance [16]. Compared with those under the impact energy of 10 J, the impact damage of the interlayers further increases and the circular deletion of elements from the middle layers occurs due to the high impact energy of 15 J.

In order to investigate the damage characteristics of specimens in the thickness direction, the stress and deformation of specimens under different impact energies obtained from the simulation results are compared with the experimental results obtained from ultrasonic A-scan tests, which are presented in Figure 20. It can be seen that the simulation results of the zone of stress and deformation for specimens under different impact energies are consistent with the experimental test, verifying the accuracy of the finite element modeling. With the increase in impact energy, the impact damage area obtained from simulation results and experimental results is increased, and obvious interlayer damage can be observed on all specimens under the high impact energy of 15 J. The zone of stress and deformation is focused on the two sides of all specimens under low impact energy, and spreads throughout the entire thickness direction of all specimens with the increase in impact energy. Furthermore, the zone of stress and deformation of specimen HL2 with a helical angle of 12.8° along the thickness direction is lowest under all impact energies and displays a non-uniform distribution along the thickness direction under high impact energy, while the zone of stress and deformation of specimen QI1 with a helical angle of 0° along the thickness direction is the largest under all impact energies and presents a uniform distribution along the thickness direction under high impact energy. This is because the helical layup structure of fiber fabrics can impede the stress transmission and crack propagation in the thickness direction of the specimen, and improve the impact resistance. Under the high impact energy of 15 J, the impact damage area of specimen HL2 is lower than that of specimens HL1 and HL3, indicating that an appropriate angle of the helical layup can sufficiently enhance the impact resistance of the composite material.

## 5. Conclusions

In this paper, bio-inspired helicoidal layups are designed to enhance the impact resistance capacity of plain woven carbon fiber fabric reinforced composite laminates. Low velocity impact tests and finite element simulation are carried out to investigate the effect of rotation angle of the helicoidal layups on the impact damage behaviors of composite laminates, including impact force response, energy absorption characteristics and damage mechanism. Based on the studies, the following conclusions are drawn:

- The numerical simulation results of impact force–time response, absorbed energy–time response, and damage characteristics are in good agreement with the experimental test results.
- With the increase in impact energy, the maximum values of impact force are increased. Under all impact energy conditions, the maximum value of impact force for specimen HL2 (rotation angle of 12.8°) is higher than that of the other rotation angle specimens, while the maximum value of impact force for specimen QI1 (rotation angle of 0°) is the lowest.
- With the increase in impact energy, the absorbed energy for all specimens is increased, as well as the energy absorption ratio. Under the low impact energy of 5 J, the energy absorption ratio is ranked in the order: HL1 > QI2 > HL3 > QI1 > HL2, while the energy absorption ratio is ranked in the order: QI1 > HL3 > HL1 > QI2 > HL2 under the high impact energies of 10 J and 15 J.
- Under all impact energies, the impact damage of specimens with helicoidal layups are lower than that of specimen QI1 (rotation angle of 0°), indicating that the helicoidal layup of carbon fiber fabric can sufficiently enhance the impact resistance of the composite material. Furthermore, the impact resistance of specimen HL2 (rotation angle of 12.8°) is the best, because of its lowest impact damage and highest impact force under all energies.

## Figures and Tables

**Table 1:** Detailed configurations of helicoidal layups.

Designation	No. of Plies	Type	Stacking Sequence
Quasi-isotropic-1 (QI1)	16	Quasi-isotropic	[0°]_16_
Quasi-isotropic-2 (QI2)	16	Quasi-isotropic	[−45°/0°/0°/45°]_4_
Helicoidal-linear-1 (HL1)	16	Helicoidal (6°)	[0°/6°/12°/…/78°/84°/90°]
Helicoidal-linear-2 (HL2)	16	Helicoidal (12.8°)	[0°/12.8°/25.6°/…/64°/76.8°/90°]_2_
Helicoidal-linear-3 (HL3)	16	Helicoidal (30°)	[0°/30°/60°/90°]_4_

**Table 2:** Mechanical properties of plain woven carbon fabric prepreg.

Property	Value
Longitudinal modulus, *E*_11_ (GPa)	36.1
Transversely modulus, *E*_22_ (GPa)	36.1
Out-of-plane modulus, *E*_33_ (GPa)	8.71
Shear modulus, *G*_13_ = *G*_23_ (GPa)	3.27
Shear modulus, *G*_12_ (GPa)	3.65
Poisson’s ratio, *υ*_13_ = *υ*_23_	0.33
Poisson’s ratio, *υ*_12_	0.06
Longitudinal tensile strength, *S*_1T_ (MPa)	460
Longitudinal compressive strength, *S*_1C_ (MPa)	250
Transverse tensile strength, *S*_2T_ (MPa)	460
Transverse compressive strength, *S*_2C_ (MPa)	250
Out-of-plane compressive strength, *S*_3C_ (MPa)	60

**Table 3:** Parameters of impact tests.

Sample Dimensions (mm)	Impact Energy (J)	Impact Velocity (m/s)	Impact Mass (kg)
120 mm × 120 mm × 3 mm	5	2.24	4
	10	3.16	4
	15	3.87	4

**Table 4:** Comparison of the maximum values of the impact force obtained from the numerical and experimental results.

Impact Energy (J)	Sample	Experimental Maximum Values of Impact Force (kN)				Numerical Maximum Values of Impact Force (kN)	Average Error (%)
		Test 1	Test 2	Test 3	Average		
5	QI1	3.15	3.32	3.19	3.22	3.39	5.3
	QI2	3.25	3.22	3.25	3.24	3.94	17.8
	HL1	2.95	2.88	2.93	2.92	3.81	23.3
	HL2	3.33	3.28	3.32	3.31	3.97	16.7
	HL3	3.33	3.25	3.26	3.28	3.95	17.0
10	QI1	3.52	3.62	3.48	3.54	3.50	1.1
	QI2	4.51	4.49	4.50	4.50	4.23	6.0
	HL1	3.83	3.83	3.92	3.86	3.61	6.5
	HL2	4.65	4.61	4.63	4.63	4.48	3.2
	HL3	4.40	4.39	4.35	4.38	4.31	1.5
15	QI1	3.55	3.54	3.56	3.55	3.92	10.4
	QI2	4.43	4.52	4.58	4.51	4.64	2.9
	HL1	4.03	3.96	3.98	3.99	4.25	6.5
	HL2	5.20	5.18	5.16	5.18	4.94	4.6
	HL3	4.55	4.46	4.52	4.51	4.49	0.2

**Table 5:** Comparison of the absorbed energy obtained from the numerical and experimental results.

Impact Energy (J)	Sample	Experimental Absorbed Energy (J)				Numerical Absorbed Energy (J)	Average Error (%)
		Test 1	Test 2	Test 3	Average		
5	QI1	0.96	0.95	1.03	0.98	0.82	16.3
	QI2	1.12	1.21	1.21	1.18	1.38	16.9
	HL1	1.47	1.49	1.48	1.48	1.50	1.3
	HL2	0.92	0.90	0.94	0.92	0.74	19.5
	HL3	1.01	0.99	1.12	1.04	0.86	17.3
10	QI1	6.52	6.73	6.82	6.69	5.17	22.7
	QI2	3.81	3.78	3.87	3.82	3.39	11.2
	HL1	3.89	3.90	3.85	3.88	3.55	8.5
	HL2	3.13	3.01	2.98	3.04	2.85	6.2
	HL3	4.19	4.15	4.20	4.18	4.10	1.9
15	QI1	12.51	12.45	12.48	12.48	11.15	10.7
	QI2	11.28	11.33	11.35	11.32	9.37	17.2
	HL1	11.59	11.59	11.56	11.58	9.58	17.3
	HL2	10.70	10.60	10.65	10.65	8.46	20.6
	HL3	11.68	11.75	11.80	11.74	9.70	17.40

## Data Availability

The original contributions presented in this study are included in the article. Further inquiries can be directed to the corresponding author.
